# Exploring the Online Health Information-Seeking Behavior in a Sample of Italian Women: The “SEI Donna” Study

**DOI:** 10.3390/ijerph19084745

**Published:** 2022-04-14

**Authors:** Loredana Covolo, Miriam Guana, Guglielmo Bonaccorsi, Laura Brunelli, Silvana Castaldi, Antonella De Donno, Alessandra Mereu, Marco Verani, Umberto Gelatti

**Affiliations:** 1 Department of Medical and Surgical Specialties, Radiological Sciences and Public Health, Section of Public Health and Human Sciences, University of Brescia, Viale Europa, 11, 25123 Brescia, Italy; umberto.gelatti@unibs.it; 2 Department of Clinical and Experimental Sciences, School of Midwifery, University of Brescia, 25123 Brescia, Italy; miriam.guana@unibs.it; 3 Department of Health Sciences, University of Florence, 50134 Florence, Italy; guglielmo.bonaccorsi@unifi.it; 4 Department of Medicine, University of Udine, 33100 Udine, Italy; laura.brunelli@uniud.it; 5 Quality and Accreditation Unit, Friuli Centrale Healthcare University Trust, 33100 Udine, Italy; 6 Department of Biomedical Sciences for Health, University of Milan, 20133 Milan, Italy; silvana.castaldi@policlinico.mi.it; 7 Fondazione IRCCS Ca’ Granda OMP Milan, 20122 Milan, Italy; 8 Department of Biological and Environmental Sciences and Technologies, University of Salento, 73100 Lecce, Italy; antonella.dedonno@unisalento.it; 9 Department of Medical Sciences and Public Health, University of Cagliari, 09042 Cagliari, Italy; alssamer359@gmail.com; 10 Department of Biology, University of Pisa, 56127 Pisa, Italy; marco.verani@unipi.it

**Keywords:** online health information seeking, women, health literacy, eHealth literacy, survey, healthcare workers, public health

## Abstract

There is much discussion about the skills of people in understanding and managing online health information. The Italian survey “SEI Donna” aimed to investigate perceptions and use of the web in women regarding health issues considering their health literacy (HL) and healthcare skills. We used an online questionnaire to explore different aspects of online health-related information-seeking behavior. The study participants (*n* = 7027) were categorized into healthcare workers (HW), healthcare students (HS), and non-healthcare women (non-HW). Half the sample (52%) searched online for a second opinion after the medical examination without statistical difference among HW, HS, and non-HW. Women in the age range of 26–40 years (OR = 1.28, *p* < 0.001), having chronic illness (OR = 1.48; *p* < 0.001), and being moderately (OR = 1.58; *p* < 0.001) or not satisfied (OR = 2.04; *p* < 0.001) with healthcare professionals were more likely to use the Internet to seek medical insight. Overall, 34% of women had a functional HL, the same being higher in HW (64%) and in HS (43%) than the rest of the women (18%) (*p* < 0.0001). The suboptimal HL suggests the need to improve HL in the general population to be skilled in surfing the web and, at the same time, to reorganize health training to improve the HL of healthcare professionals, also enriching their communication skills.

## 1. Introduction

Health information-seeking behavior has become increasingly common over the last few years, especially thanks to the advent of the Internet and the spread of social media [1]. Women seem to be more active in looking for health-related information online than men [2,3,4,5,6]. Data from an Italian report showed a significant increase in Internet use since 2011, with women overtaking men [7]. At the same time, a recent European report [1] showed that women use online social networks more frequently than men do and, additionally, according to a systematic review on the topic [8], women are more active than men in seeking information on lifestyle and health, suggesting a higher attention to health and disease prevention among women. Other studies on women highlighted that poor health and the presence of chronic diseases are predictors of online health information-seeking behavior [2,6,9]. Furthermore, it was shown that people went online primarily for reassurance or for a second opinion and women were more likely than men to seek help for someone else, as well as for both themselves and others [9]. In a study focused on the difference in health-seeking behaviors, men appeared to be more concerned with the comprehensiveness and accuracy of the information, whereas women demonstrated greater interest in cognition, such as the ease with which they can read and understand the information. At the same time, it was shown that women more than men were shown to be more likely to consult a wide range of sources of information after a first consulting: from other health professionals to family and friends [10].

From these points of view, the Internet offered a variety of information sources (institutional and non-institutional, communities, and individuals) with different levels of accuracy and quality with a high probability of facing misinformation and disinformation [11]. Of note, a recent survey carried out by the Italian Communications Authority [12] showed that, despite the high demand for scientific information, a very low supply and level of expertise among journalists dealing with scientific issues (less than 10% of them) was recorded, leading to a dissemination of inaccurate information. In this regard, health literacy (HL), defined as “an individual’s ability to acquire, process, and understand basic health information and services in order to make appropriate health decisions” [13], is a skill that has become very topical. In fact, HL has long been associated with those indicators of health becoming increasingly important in predicting health promotion and prevention [14,15]. Specifically, HL has been shown to influence health decisions of women in different period of their lives, from pregnancy to children’s care, cancer screening adherence, and therapies [16]. At the same time, it has been shown that the trustworthiness of health-related information sources depends on HL as people with limited HL are more likely to use and trust Web sources containing lower quality health information against data coming from healthcare professionals [17]. Therefore, in a context where the main source of health information is the Web, the ability to properly use the emerging information and communications technologies, i.e., e-HL, seems to be necessary to protect individuals from inaccurate and potentially dangerous information for their health [18]. Many instruments have been developed so far to measure objectively the ability to understand medical information, or to allow a HL self-assessment [19,20]. Most of these studies measure HL using one tool and mainly targeting the general population or patients [21,22,23,24], and only a few studies focused on healthcare workers [24,25]. While from the individual’s point of view the ability to understand written health information is important to make decisions about health promotion, disease prevention, health care maintenance, and to navigate the healthcare system [26], from the health professional’s point of view HL is also important for the ability to discuss health with patients. In fact, over recent decades, healthcare professionals have found themselves managing patients who are more and more informed through the Internet, making this aspect even more crucial from the perspective of the patient–doctor relationship.

In this context, the aims of this survey were: (1) to investigate overall perceptions and use of web resources in women regarding health issues, (2) to estimate the level of trust women had in the information found on different Internet resources, and (3) to assess any differences in health information-seeking behaviors, taking into account the users’ HL and healthcare skills.

The need to be able to search and use health information properly has become crucial during the recent COVID-19 pandemic [27,28], a period characterized by an overwhelming dissemination of information, disinformation, and misinformation. This study, carried out just before the pandemic, led to a reflection on possible factors influencing the disorientation of both healthcare professionals in managing communication and the population in understanding the correct information.

## 2. Materials and Methods

### 2.1. Recruitment

From February to July 2019, we carried out a cross-sectional study in Italy using an online self-administered survey. The acronym of the study was “SEI Donna-Salute E Internet per la Donna” (Health and Internet for women). Participation in this survey was voluntary and completely anonymous, without any possibility to trace back who filled in the questionnaire. The purposes of the survey were clearly described in the first part of the questionnaire. Participants had the option to withdraw consent at any time prior to submitting responses. Inclusion criteria were female gender, age ≥18 years old, and use of the Internet to seek health information. According to Italian law [29], this study did not require approval by the Ethics Committee. However, the protocol was sent to the local Ethics Committee of Brescia for acknowledgement.

The survey was designed using the software LimeSurvey (LimeSurvey GmbH, Ham-burg, Germany) and it was made accessible via a link disseminated through different channels: institutional mailing lists, social media such as Facebook, Instagram, and WhatsApp, and all social media pages dealing with women health. Participants could also see the link on the website www.ondaosservatorio.it (accessed on 11 April 2022)., a website focused on women’s health; www.syrio.org (accessed on 11 April 2022), the website of Italian Society of Obstetrical-Gynecological-Neonatal Sciences (SYRIO); and the website of an online public health newspaper. In all cases, it was asked that the survey link be re-shared on different platforms for snowball sampling. The link was also distributed by engaging different scientific societies and women’s associations in the health field including voluntary associations.

The online survey was developed in accordance with the checklist for reporting results of an internet e-survey (CHERRIES) [30]. See Supplementary File S1.

### 2.2. Measurements

Overall, the questionnaire was designed with different sections (see Supplementary File S2) with the aim to understand what information was searched, how it was searched, how it was perceived, and how much women were skilled in looking for and using online information.

#### 2.2.1. Sociodemographic Information

The first question was “Have you ever used the Internet to look for health information?”. Only women who answered “yes” could go on with the questionnaire filling. Regarding both women who answered “no” and “yes”, we collected socio-demographic data including age, education level, region of residence, occupation, and marital status. We categorized respondents into three groups according to age: 18–25 years old, 26–40 years old, and >40 years old. We asked respondents whether they studied or worked in the healthcare field and, based on the question on occupation, we categorized the sample into three groups: healthcare workers (HW), healthcare students (HS), and non-healthcare women (non-HW). A 7-point Likert scale (1 = very poor, 7 = excellent) was used to assess the perceived health status. Answers were categorized as follows: “poor” scores—1 through 3 points; a “fair” score—4 points; and “good” scores—5 through 7 points. The presence of chronic diseases was assessed by a self-reported answer (yes/no).

#### 2.2.2. Web and Social Media Use

We surveyed respondents on the type of health information sought, choosing a maximum of three topics organized according to a priority order, to better understand the choices respondents deemed more important. We also asked participants what kind of social networks they used and if they were part of social media groups dealing with health issues. We asked respondents whether they navigate the Web after a medical examination and—in case of a positive answer—they were further asked to illustrate up to three reasons according to a priority order.

#### 2.2.3. Trust in Web and Health Professional Sources of Information

The degree of trust towards different web sources in addition to satisfaction with health professionals were assessed using a 7-point Likert scale (1 = not at all, 7 = extremely). Answers were categorized as follows: 1 through 3 points meant “slightly”, 4 points meant “moderately”, and 5 through 7 points meant “very”. Concerning the overall satisfaction with health professionals, respondents who rated a score of 5 or less were asked to indicate a maximum of three critical issues classified according to a priority order. The trustworthiness of health-related information retrieved from websites was investigated by asking women to indicate the website they thought to be more reliable from a list of selected resources including one institutional website (website of the Italian Ministry of Health), and five non-institutional websites with content related to women’s health. The impact of online health information was assessed through the following questions: “Do you think online information improved your health knowledge?” and “Do you think online information influenced your health habits/decisions?”. We asked women to assign a score according a 7-point Likert scale (1 = not at all, 7 = extremely). Answers were categorized as follows: 1 through 3 points meant “not at all/slightly”, 4 points meant “moderately”, and 5 through 7 points meant “very/extremely”.

#### 2.2.4. Health Literacy

We measured HL using the Italian validated version of three validated questionnaires, namely the single-item literacy screener (SILS-IT) [31], Medical Term Recognition Test (I-METER) [23], and e-HL Scale (IT-eHEALS) [32]. The SILS-IT consisted of a single question: “How often do you need to have someone to help when you read instructions, pamphlets, or other written material from your doctor or pharmacy?”. Possible responses were “never” (1), “rarely” (2), “sometimes” (3), “often” (4), and “always” (5). Scores >2 indicate some difficulty with reading printed health-related material. I-METER is a questionnaire composed of 40 medical words and 30 non-words, i.e., pretend medical words that sound like medical terms. Respondents were asked to mark words they recognize as real medical terms. HL skills were defined as the number of words correctly recognized, with higher scores reflecting higher HL. Scores of 0–20 indicate low literacy, scores of 21–34 indicate marginal literacy, and scores of 35–40 indicate functional HL [33]. The IT-eHEALS determines consumers’ combined knowledge, confidence, and perceived skills in finding, evaluating, and applying electronic health information to health problems. The measure consists of 8 items scored on a 5-point Likert scale ranging from 1 (strongly disagree) to 5 (strongly agree). Higher scores on the IT-eHEALS indicate higher eHealth literacy (total score range = 5–40).

### 2.3. Statistical Analysis

The analyses included descriptive statistics (i.e., frequencies and percentages for categorical variables and mean values with standard deviations for continuous variables). Comparisons between groups were made using the χ^2^ test or Fisher’s exact probability test for categorical variables and Mann–Whitney test for continuous variables. A binary logistic regression model was carried out with online health information seeking after a medical examination as the dependent variable. The covariates to be included into the final model were selected using a backward selection process, with a univariate *p* < 0.05 as the main criterion. To check for collinearity among variables, the Spearman correlation test was used. The results of logistic regression have been reported with adjusted odds ratios and 95% confidence intervals. A *p*-value less than 0.05 was considered as statistically significant for all analyses. I-METER and IT-eHEALS were tested for reliability using the Cronbach’s alpha for both “real terms” and “non-realm terms”. Values higher than 0.8 indicate good or excellent internal consistency, while values between 0.7 and 0.8 indicate acceptable internal consistency. Statistical analyses were performed using STATA (Stata Statistical Software: Release 14.0 College Station, TX, USA: Stata Corporation).

## 3. Results

### 3.1. General Characteristics of Sample

Among the 7296 respondents who completed the survey, 269 (4%) did not use the Internet to seek health information. Non-seekers of online health information were older than seekers (41.7 ± 16.8 vs. 34.1 ± 13.4, *p* < 0.0001). No difference was found between non-seekers and seekers according to education level (degree: 48.0% vs. 50.0%, respectively; *p* > 0.05) and area of residence (63.6% of non-seekers lived in the north of Italy, 17.1% in central Italy, and 19.3% in the south). Among non-seekers there were less students (26% vs. 35%, *p* = 0.004) and more retired people compared to online seekers (8% vs. 2%, *p* < 0.0001). In Table 1, socio-demographic characteristics of online seekers included in the study (*n* = 7027) are shown.

Overall, the mean age was 34.1 ± 13.4. Fifty percent of the sample had a degree. Most women lived in the north of Italy (64%) and were married or cohabiting (56%). Women working or studying in the healthcare field made up 42% of the respondents (*n* = 2991), being 25% and 17%, respectively, of the sample. Thirty-seven percent of women had children, who were at pediatric age in 60% of them (*n* = 1583) and 23% of all women. Most women (84%) self-evaluated their health as from good to excellent. Thirteen percent of them (*n* = 937) reported having a chronic illness.

### 3.2. Online Seeking Behavior Characteristics

In Table 2, online health information-seeking behavior and perceptions are shown with a comparison among non-HW, HS, and HW.

**Figure 1:**
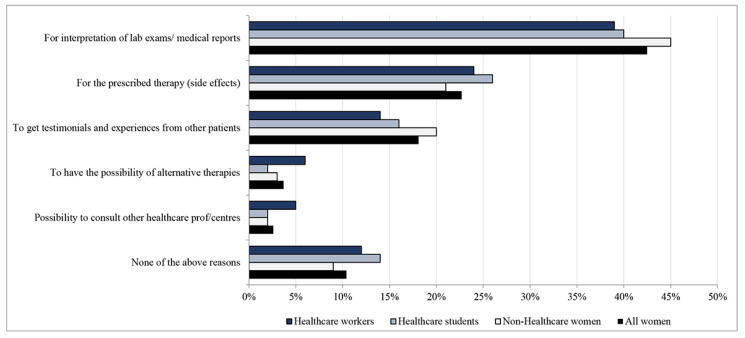
Distribution of responses regarding the kind of information searched by women using Internet to get a second opinion after medical examination (*N* = 3674).

One-third of women (*n* = 2328, 33%) reported to follow some health-related groups on social media. HW and non-HW followed groups mainly on Facebook (87% and 67%, respectively), differently from HS (55%) who mainly followed groups on Instagram (68%) compared to non-HW (38%) and HW (20%) (data not shown in table). Thirty-three percent of non-HW purchased health-related products online. In particular, cosmetics (75%), supplements (42%), and herbal products (20%) were purchased by women without statistical difference among the three groups. Non-HW purchased drugs and homeopathic products more than HW/HS (16% vs. 11%, *p* = 0.002, and 10% vs. 7%, *p* = 0.005, respectively) (data not shown in table). Overall, lifestyle (31%) followed by information on specific diseases (25%) and body care/aesthetics (17%) were the three topics mainly searched online. The order of the first position items did not change throughout the three groups except when analyzing HW, whose third topic was specialists/hospitals seeking. More than half of the women (52%) used the Internet after being checked up by a healthcare professional without statistical difference among the three groups. About one-third of the sample thought that online information greatly improves their health (35%). Similarly, all women reported online information as influencing their health habits/decisions at the level of “very/extremely” (34%). With regards to the level of trust in the source of information, institutional websites reached the highest level from the majority of all women (80%) compared to other sources. Additionally, when women were asked about the trustworthiness of health information reported by specialized doctors and general practitioners, most of them indicated a high score. In particular, 97% (*n* = 1119) of HS, followed by 95% (*n* = 3697) of non-HW and 92% (*n* = 1624) of HW, trusted the specialized doctor at the level of “very/extremely”. Non-HW (79%, *n* = 3202) trusted their general practitioners at the level of “very/extremely”, followed by HS (78%, *n* = 945) and HW (75%, *n* = 1330) (data not shown in table). In general, half of the sample (54%) identified the Italian Ministry of Health website as the most reliable, particularly by HW (68%), followed by HS (60%) compared to non-HW (45%), *p* < 0.0001, even if 30% of women were unable to respond, mainly non-HW, *p* < 0.0001 (Table 2). Regarding the reasons why women went online after being checked up by a healthcare professional, the majority of them stated the need to get more detailed information as the main reason for their search (*n* = 2455, 67%); the second reason was that the information given was not comprehensive (*n* = 691, 19%). There were no statistical differences among the three groups. The main topics of these information searches are reported in Figure 1.

Interpretation of lab exams/medical reports was indicated less by non-HW (39%) compared to HS (40%, *p* = 0.008) and HW (45%, *p* = 0.02). With regards to information on the prescribed therapy, there was a statistical difference between non-HW and HS (21% vs. 26%, *p* = 0.006). The need to search for experiences from other patients was reported more frequently by non-HW (20%) compared to HS (16%, *p* = 0.02) and HW (14%, *p* < 0.0001).

### 3.3. Satisfaction with Health Professionals

**Figure 2:**
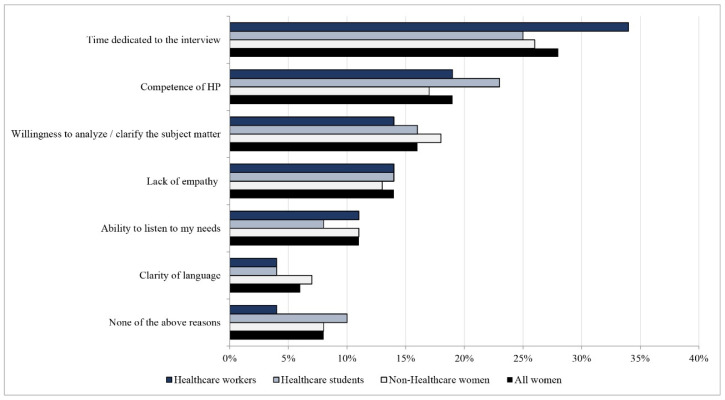
Distribution of the most critical issues reported by women not completely satisfied with healthcare professionals (*N* = 4414).

Concerning the overall satisfaction with healthcare professionals, 78% the sample was very/extremely satisfied, particularly HW (81%, *n* = 1449), compared to HS (79%, *n* = 950) (*p* > 0.05) and non-HW (77%, *n* = 3046) (*p* < 0.0001). For all women, the most critical issues were time dedicated to the medical interview (28%), the competence of healthcare professionals (19%), and willingness to clarify the subject matter (17%) (Figure 2).

Time dedicated to the medical interview was indicated as a concern by HW (34%, *n* = 365) more often than HS (25%, *n* = 190) and non-HW (26%, *n* = 667) (*p* < 0.0001). The competence of healthcare professionals was indicated more by HS (23%) compared to both HW (17%, *p* < 0.0001) and non-HW (19%, *p* = 0.02). With regards to the third item, willingness to clarify the subject matter, there was a statistical difference between non-HW and HW (18% vs. 14%, *p* = 0.008).

### 3.4. Health Literacy

Total score of the IT-eHEALS ranged from 10 to 40 with a mean of 26.7 ± 6.8. When comparing women working/studying in the healthcare field with the rest of the sample, the first group (HW) showed a significantly higher IT-eHEALS score (respectively, 30.2 ± 6.4 vs. 28.1 ± 6.2 vs. 24.7 ± 6.4, *p* < 0.0001) (Table 3).

**Figure 3:**
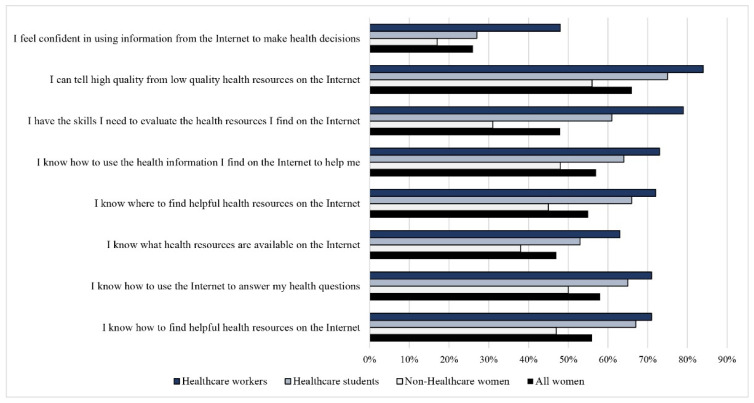
IT-eHealth Literacy Scale agreement in healthcare workers, students, non-healthcare women, and all women.

Figure 3 shows the response frequencies for the agreement of each IT-eHEALS item.

Less than 50% of HW (48%), about one-third of HS (27%), and only 17% of non-HW agreed/strongly agreed with the statement “I feel confident in using information from the internet to make health decisions”. Except for the previous statement, more than 60% of HW agreed or strongly agreed with all the other IT-eHEALS statements. Similar results were found in case of HS, albeit at a lower percentage than HW, although the difference was statistically significant for each statement (*p* < 0.05). Only in the case of the statement “I know what health resources are available on the internet” HS agreed with a lower percentage (53%). Just over half non-HW (56%) agreed/strongly agreed with the statement “I can tell high quality from low quality health resources on the Internet”. Less than 50% agreed/strongly agreed with all the other statements with difference statistically significant compared to both HS and HW (*p* < 0.0001). As shown in Table 3, according to I-METER, the overall frequency of women with functional HL was 34%, the same being higher in HW (64%) and in HS (43%) than the rest of the women (18%). The difference was statistically significant also when comparing HW with HS (*p* < 0.0001). Based on the Italian single-item literacy screener (SILS-IT), findings evidenced that 28% of women (*n* = 1945) indicated some difficulty with reading printed health-related material (Table 3). Low HL, as assessed with SILS-IT was significantly higher in women not involved in the healthcare field (34%) compared to the HS (24%) and HW (16%). Similar to what has been found for the I-METER, the difference was statistically significant also comparing HW with HS (*p* < 0.0001). The same results were found taking into consideration the IT-eHEALS scores. Overall, functional literacy was higher among women who stated to trust institutional websites at the level of “very/extremely” compared to the rest of the women (36% vs. 24%, *p* < 0.0001). At the same time, women who stated to trust institutional websites at the level of “very/extremely” had a mean IT-eHEALS score statistically higher (27.5 ± 6.6) than those who stated to moderately trust institutions (23.8 ± 6.7, *p* = 0.002) and slightly/not at all trust them (vs. 22.8 ± 6.7, *p* = 0.0001) (data not shown in table). I-METER had a high internal consistency both for the real term group (alpha = 0.93) and non-real term group (alpha = 0.81). A high internal consistency was found also on IT-eHEALS (alpha = 0.91). Even if e-HEALS scores weakly correlated with I-METER scores (r = 0.35, *p* < 0.0001) and SILS-IT (r = −0.31, *p* < 0.0001), the mean IT-eHEALS score was lower among women with low/marginal literacy on the I-METER (25.1 ± 6.5) than women with functional literacy (29.8 ± 6.3) (*p* < 0.0001). Similarly, the mean IT-eHEALS score was lower among women with low literacy on SILS (24.0 ± 6.1) than women with high literacy (27.7 ± 6.8) (*p* < 0.0001). The Spearman’s coefficient was also low regarding the I-METER and SILS-IT scores (−0.24, *p* < 0.0001). However, the correlation between the two scores was statistically significant considering both the mean and HL categories. The mean I-METER score was low among women with low literacy on the SILS (28.0 ± 8.2) compared with the high literacy category (31.2 ± 7.4) (*p* < 0.0001). Functional HL according to I-METER was higher among women with high literacy on the SILS-IT than among women with low literacy (39% vs. 19%, *p* < 0.0001).

### 3.5. Predictors of Online Health Information Seeking after Medical Examination

All the factors shown in Table 4, except for I-METER, were statistically associated with health information seeking after medical examination in the univariate analysis and therefore included in the logistic model.

Women in the age range 26–40 were more likely to seek health information (OR = 1.3) independently from their education level. Women with chronic illness (OR = 1.5) and those moderately (OR = 1.6) or not satisfied (OR = 2.0) with healthcare professionals were more likely to use the Internet to seek medical insight. Following health groups on social media (OR = 1.6), thinking that online information improves (OR = 2.2) or influences health decisions (OR = 1.7) were all factors associated with searching information online after a medical examination. Low HL measured according to SILS-IT was significantly associated with the search for a second opinion (OR = 1.4), although this was not confirmed when considering low HL evaluated according to I-METER. Health information-seeking behavior was slightly associated with a higher IT-eHEALS score.

## 4. Discussion

Online health information seekers recruited through this survey were mainly young women, highly educated, having a good health, and particularly interested in topics related to lifestyles. Characteristics were similar to those found more than ten years ago in Italy [2] and in other countries such as Finland [3], Australia [6], the UK [9], and Germany [34], where gender determinants and patterns of online information-seeking behavior were investigated. However, this is the first study, to our knowledge, carried out in Italy in a large sample of women exploring differences in web use, attitude, and perception taking into account HL and healthcare expertise.

### 4.1. Web Use and Women’s Perception

The main results of this survey highlighted a large use of the Internet as a tool to search for health information among Italian women, mainly after a medical examination. Half of the sample stated to use the Internet for a second opinion, and the need to have more detailed information was the main reason in 67% of the subjects, without any difference among HW, HS and non-HW. The need to have further information after a medical check-up might be due to poor health as well as patient–doctor relationships not always up to expectations, as confirmed by the multivariate analysis, and as also found in the literature [6,33]. Findings of overall satisfaction with healthcare professionals showed that most women (64%) gave a score of 5 or less on a 7-point Likert scale. The first critical issue reported was the time dedicated to the interview. The perceived need for extra time might depend on the need to have more information or more in-depth explanations about medical exams, but also on the need for comfort, confirming previous findings [9]. Time as a critical issue was largely highlighted by HW than non-HW. HW probably needed to discuss more with health professional by virtue of their knowledge and probably require having more detailed information. Nevertheless, willingness to clarify the subject matter in addition to the ability to listen to one’s needs and lack of empathy were reported as the second main reason by 68% of HW without any differences compared to HS (69%) and non-HW (70%). In fact, nowadays, in the organization of healthcare services time devoted to patients is more and more restricted. The clinical time is preserved although not in the same way as the time dedicated to listening and support [35].

The need for support to read lab examinations followed by getting testimonials and experiences by other patients was the main reason why women went online after medical examinations and underlined the fact that the time devoted to patients cannot be merely clinical as emphasized in a systematic review on the doctor–patient relationship by Derksen et al. [36]. While in some cases, online health information seeking may be a consequence of an unsatisfactory doctor–patient relationship, it can also be seen as a challenge. As highlighted by a recent systematic review [37], the need to go online to search for health information should be considered as positive in terms of patient–doctor relationship, where trust in health institutions and particularly in healthcare professionals as individuals is maintained [11,38]. The findings of our survey seem to confirm the results highlighted by this systematic review. Healthcare professionals could therefore take advantage of new communication technologies to address patients’ needs, for example interacting with them via online healthcare communities [39] and/or guiding them towards reliable healthcare web channels (websites or social media groups). This would help fill in the gap of lack of time in the clinical setting, but also prevent patients from being misled by online misinformation and help them better manage their own health. Online health information-seeking behavior could be seen as an opportunity to have a more motivated and collaborative patient, positively influencing the doctor–patient relationship [11,37,40]. Moreover, understanding those health information-seeking preferences could help healthcare professionals improve communication with patients and further promote trust [41]. A recent study showed how online information significantly influenced parents’ trust in their pediatrician’s diagnosis and their probability of seeking a second opinion, especially when they had found contrasting information [42].

### 4.2. Level of Trust in Information Sources

The impact of the Internet is evident when considering that just over a third of the surveyed women thought that online information improved their health knowledge and particularly influenced their health-related decisions, regardless of their belonging to the healthcare field. This aspect was also a predictive factor for searching for a second opinion online after visiting the doctor, in a certain sense recalling Boyce’s research model [43] according to which, perceived usefulness had a positive influence in online seeking behavior in addition to perceived ease of use, which was a pattern that seemed to characterize women more than men [10]. In a representative European sample, the perception of the Internet as a tool to improve knowledge in health-related topics was stronger than in our survey (59%) [44]; however, similarly to the data from the Eurobarometer survey carried out in 2021, trust in the Internet and social media was quite low [1]. Just a third of Europeans (35%) said they “tend to trust” the Internet and 19% trust in social media, lower when compared to traditional media (radio, television, and written press). Similarly, in our survey, institutional websites were more trusted by the majority of women compared with non-institutional websites and social media. On the other hand, it is worrying that more than half of non-HW, but also 32% of HW, did not recognize the Italian Ministry of Health website as reliable in a list where the other were not institutional websites. This finding is surprising considering that it was also confirmed among those who stated to “trust a lot” institutional websites. In other words, they trusted institutional websites, even if they do not recognize the national Ministry of Health website as such. This result leads our discussion to HL findings.

### 4.3. Role of Health Literacy and Healthcare Skills

In a similar way to how our findings related to factors influencing the second opinion online, a recent study showed that better HL was associated with health-related information-seeking behavior [45], in addition to being female, having a graduate degree, and reporting a poor/fair health status participation in social groups. They measured HL using a tool like SILS. The authors reported that individuals with higher HL are probably more comfortable seeking out health information, are more skilled at knowing what to search for and how to find it and are more comfortable interpreting the information that they access.

Overall, using I-METER, we found that only 18% of non-HW had a functional literacy and the score of e-HL in this group was low (24.7 ± 6.4). These results are similar to those reported in previous studies [21,22,23,24] evaluating the HL on the general population. Few studies were carried out on HW’s health literacy [24,25]. For this survey, the mean IT-eHEALS score of HW (30 ± 6.4) was lower than the scores registered in HW women recruited from hospitals and health centers across Vietnam (32.8 ± 4.5) and scores found in a small sample of Italian people with studying or working experience in the healthcare field (31.9 ± 5.9). Nevertheless, HL and e-HL in HS and HW were, as expected, higher than non-HW. We cannot say that they were optimal, especially considering HL measured by an objective tool such as I-METER, which showed that 36% of HW and 57% of HS had marginal/low literacy. Considering that, differently from a performance-based instrument, a self-assessed tool may be influenced by an overestimation of personal skills, it was shown that objective HL tools measure the skills in identifying low-quality health information more efficiently than subjective HL tools, such as the e-Heals Literacy scale and SILS [46]. These findings raise the concern about the reason why a large part of women working/studying in the healthcare field had not an adequate HL. Regardless of the instrument’s type, HL assessment is based on the evaluation of general rather than technical skills. This fact suggests a reflection on health training courses that may be too specialized. Of course, inadequate literacy is not just a problem of the individual HW or HS but of the system (organization/institution) which probably must rethink its healthcare training, providing HL tools and improving communication skills. Interestingly, it has been shown that HL awareness training for HW improved healthcare professionals’ HL awareness and communication skills with patients [28,47]. In fact, clear and effective communication is even more important, especially when dealing with people with low literacy [16].

### 4.4. Practical Implications in the COVID-19 Pandemic Scenario

In a context where there are a lot of health information, HW are assumed to play a reference role for the public. However, the question is what happens if HW themselves do not possess an adequate HL? The findings of this survey, carried out pre-COVID-19 pandemic, suggest the possibility that a low HL partially explains, on the one hand, the inability of healthcare staff to manage effectively communication and, on the other hand, the difficulties of the general population when navigating the overwhelming amount of information available through the media. Of course, the health emergency led people to the need to be constantly informed; people are nowadays hungry for news, especially science-related news as already highlighted by an Italian report published before COVID-19 pandemic [10]. The infodemic phenomenon, i.e., the rapid spread on misinformation through the Web due to the COVID-19 pandemic, showed how skills in reading, evaluating, and properly using online health information are very crucial [27,48,49]. It is interesting to note that, even before we knew what COVID-19 was, Larson, the founding director of the Vaccine Confidence Project [50], warned the scientific community against viral misinformation, calling it the greatest pandemic risk. It is increasingly evident, therefore, that individuals should protect themselves against unreliable information by being equipped with health information literacy. To this purpose, an approach based on neutralizing misinformation before it occurs through the explanation of argumentation techniques used in the misinformation has been shown to be effective [51]. In addition to this, continuous training and education have been recognized as effective approaches to improving healthcare workers’ HL, further improving health care delivery, communication, shared decision making, and patient health outcomes [28,39]. This sort of training would be necessary in the event of the employment of digital channels as an opportunity to guide the public towards verified sources of online health information and to fill in the gap in the doctor–patient relationship, fostering also a trust in healthcare professionals [52,53,54]. Social media has contributed to disintermediation, i.e., information without intermediaries, a phenomenon closely linked to the Internet, to apomediation through engagement and interactions [35,55,56]. The ability to successfully navigate the Web, especially for healthcare professionals, is therefore crucial for contributing to the apomediation process, learning to play a role as a guide or interpreter, while individuals continue to improve their information-seeking skills [35]. Overall, it is now necessary to identify the most effective strategies and interventions to empower individuals’ critical thinking skills, i.e., critical HL [57]. This also means rethinking educational programs that can be implemented on different levels of education and in different contexts, through a shared effort that requires different competences [49,57].

### 4.5. Strength and Limitations

The strengths of our study are its relatively large sample size and the assessment of validated health literacy using either performance-based tools (I-METER) and self-assessed measures (SILS-IT and IT-eHEALS) as suggested until a comprehensive HL measurement will be available [19,20]. To our knowledge, few studies analyzed HL on HW and the large number of women working/studying in healthcare field led to highlight some important considerations on this issue, also from the perspective of the pandemic context. In fact, conducting the study before the pandemic allowed us to reflect on the possible critical issues of a population impacted by the infodemic.

This study also presents some limitations. One major limitation, as an online survey, was the convenient sampling that may limit the generalizability of the findings. The use of different communication channels in different Italian regions might have minimized the selection bias due to the source of dissemination of the survey. The prevalence of women either working or studying in the health sector could have been higher considering the main source of the survey link dissemination, and instead most of the sample was characterized by non-HW. The little percentage of women who did not use the Internet to seek health information (4%) might represent another potential selection bias. However, according to ISTAT (National Institute of Statistics), people under 55 years old who never use the Internet amounted to less than 10% in 2019 [58], the year of data collection. On the other hand, the large sample of women who use the Internet and seek health information online represents, in fact, the target population of this survey. Data from ISTAT 2019 reported that around 90% of women aged 18–44 used the Internet every day, the age range represented by 76% of our sample. In addition, data from a European survey in 2014 [44] showed that 59% of Italian people used the Internet to seek health-related information.

The voluntary participation to the survey may lead to another possible selection bias. Women more sensitive to health topics or who were perceived to be more skilled in using the Internet might be more motivated to participate. The sample was also characterized by a high percentage of women with a university degree. This was a selection bias, however, that reinforced the result of the overall non-optimal HL, in the sense that the percentage of low HL was probably underestimated. Moreover, the sample had a high percentage of young women in good health: women who are likely to go to the doctor less frequently and likewise seek health information online; however, they are the ones who in the future will increasingly access health information through digital tools. Finally, data about the specific area of expertise of HW were not collected; therefore, it is difficult to understand the influence of different training and professional profiles on HL. In any case, we found that HS had HL and IT-eHEALS scores higher than the general population, highlighting the role of health training.

## 5. Conclusions

The suboptimal HL evidenced by the survey suggests the need to improve HL in the general population in order to be “inoculated” against misinformation and, at the same time, to reorganize health training in order to improve the HL of healthcare professionals and, consequently, enrich their communication skills. In fact, the more dissatisfied patients are with their doctor, the more they look for information online with the risk of being more exposed to negative consequences of misinformation in case of low HL. The Internet is a fantastic tool because it allows everyone to access a lot of information in a short time; however, “besides food you need teeth”, meaning that patients, and in wider terms citizens, need HL to critically manage health information. At the same time, from the perspective of the doctor–patient relationship, healthcare professionals need to equip themselves with a framework to respond effectively to their patients’ needs, in order to establish trust and avoid possible exposure to misinformation.

## Figures and Tables

**Table 1:** Characteristics of online seekers.

Characteristics	*N* = 7027 *N* (%)
Age (Years)	
18–25	2534 (36.1)
26–40	2431 (34.6)
>40	2062 (29.3)
Education	
Less than high school graduate	292 (4.2)
High school graduate	3218 (45.8)
Degree	3517 (50.0)
Area of residence in Italy	
North	4459 (63.5)
Middle	1210 (17.2)
South and Islands	1358 (19.3)
Status of employment	
Employed	3030 (43.6)
Freelance professional	748 (10.5)
Student	2449 (34.9)
Full-time homemaker/Unemployed	610 (8.7)
Retired	160 (2.3)
Work/study in healthcare field	
Students	1207 (17.2)
Workers	1784 (25.4)
Marital status	
Single	2762 (39.3)
Cohabitant/married	3934 (56.0)
Separated/divorced	270 (3.8)
Widow	61 (0.9)
Maternity status	
Pregnant	231 (3.3)
Women with children	2612 (37.2)
Women with children aged 0–14 years	1583 (22.5)
Health status	
Good/Excellent	5923 (84.3)
Fair	795 (11.3)
Very poor/poor	309 (4.4)
Presence of chronic illnesses	
Yes	937 (13.3)

**Table 2:** Online health information-seeking behavior and perceptions in non-healthcare vs. healthcare workers/students.

Items *n* (%)	All Women	Non-Healthcare Women (A)	Healthcare Workers (B)	Healthcare Students (C)	*p* Value ^1^
	*N* = 7027	*N* = 4036	*N* = 1784	*N* = 1207	
Health groups on social network followers	2328 (33.1)	1027 (25.5) ^3,4^	671 (37.6) ^5^	630 (52.2)	<0.0001
Health blogs followers	1029 (14.7)	518 (12.8) ^3,4^	281 (15.8) ^5^	230 (19.1)	<0.0001
Purchase health products online	2212 (31.5)	1312 (32.5) ^4^	600 (33.6) ^5^	300 (24.9)	<0.0001
Health-related information searched for on the Internet					
Lifestyle	2188 (31.1)	1310 (32.5)	504 (28.3)	374 (31.0)	ns
Specific disease	1752 (24.9)	994 (24.6)	430 (24.1)	328 (27.2)	ns
Body care/aesthetics	1213 (17.3)	781 (19.4) ^3^	169 (9.5) ^5^	263 (21.8)	<0.0001
Specialists/hospitals	612 (8.7)	308 (7.6) ^3,4^	245 (13.7) ^5^	59 (4.9)	<0.0001
Therapies/drugs	589 (8.4)	247 (6.1) ^3,4^	218 (12.2)	124 (10.3)	<0.0001
Alternative medicine	374 (5.3)	220 (5.5) ^3,4^	139 (7.8) ^5^	15 (1.2)	<0.0001
Health products purchase	254 (3.6)	156 (3.9)	69 (3.9) ^5^	29 (2.4)	0.04
Cosmetic medicine or surgery	45 (0.6)	20 (0.5)	10 (0.6)	15 (1.2)	ns
Use of internet after medical examination	3674 (52.3)	2141 (53.1)	898 (50.3)	635 (52.6)	ns
Belief that online information improves own health knowledge ^2^					
Not at all/slightly	2567 (36.5)	1420 (35.2) ^3,4^	693 (38.9)	454 (37.6)	0.02
Moderately	2024 (28.8)	1178 (29.2)	498 (27.9)	348 (28.8)	ns
Very/extremely	2436 (34.7)	1438 (35.6)	593 (33.2)	405 (33.6)	ns
Belief that online information influences own health habits/decisions ^2^					
Not at all/slightly	2868 (40.8)	1654 (41.0)	738 (41.4)	476 (39.4)	ns
Moderately	1765 (25.1)	1009 (25.0)	429 (24.1)	327 (27.1)	ns
Very/extremely	2394 (34.1)	1373 (34.0)	617 (34.6)	404 (33.5)	ns
Trust on health information shared by friends through social networks ^2^					
Not at all/slightly	5855 (83.3)	3243 (80.3) ^3,4^	1550 (86.9)	1062 (88.0)	<0.0001
Moderately	864 (12.3)	564 (14.0) ^3,4^	182 (10.2)	118 (9.8)	<0.0001
Very/extremely	308 (4.4)	229 (5.7) ^3,4^	52 (2.9)	27 (2.2)	<0.0001
Trust on health information from non-institutional websites ^2^					
Not at all/slightly	4398 (62.6)	2365 (58.6) ^3,4^	1275 (71.5) ^5^	758 (62.8)	<0.0001
Moderately	1735 (24.7)	1097 (27.2) ^3,4^	339 (19.0) ^5^	299 (24.8)	<0.0001
Very/extremely	894 (12.7)	574 (14.2) ^3,4^	170 (9.5) ^5^	150 (12.4)	<0.0001
Trust on health information from institutional web-sites ^2^					
Not at all/slightly	457 (6.5)	324 (8.0) ^3,4^	92 (5.2)	41 (3.4)	<0.0001
Moderately	919 (13.1)	630 (15.6) ^3,4^	156 (8.7)	133 (11.0)	<0.0001
Very/extremely	5651 (80.4)	3082 (76.4) ^3,4^	1536 (86.1)	1033 (85.6)	<0.0001
Most reliable website					
Website of Italian Ministry of Health (www.salute.gov.it, accessed on 11 April 2022)	3765 (53.6)	1822 (45.2) ^3,4^	1216 (68.2) ^5^	727 (60.2)	<0.0001
Non-institutional websites	643 (9.2)	449 (11.1) ^3,4^	133(7.4) ^5^	182 (5.1)	<0.0001
None of websites presented	501 (7.1)	251 (6.2) ^3,4^	129 (7.2) ^5^	121 (10.0)	<0.0001
Not known	2118 (30.1)	1514 (37.5) ^3,4^	306 (17.2) ^5^	298 (24.7)	<0.0001

^1^ *p* value (χ^2^ test) refers to comparison among the three groups (A, B and C); ^2^ Likert points 1-2-3 were recorded as “Slightly”; 4 as “Moderately”; 5-6-7 as “Very”; ^3^ A vs. B; *p* < 0.05; ^4^ A vs. C; *p* < 0.05; ^5^ B vs. C; *p* < 0.05; ns = not significant.

**Table 3:** Health literacy (HL) according to IT-eHEALS, I-METER, and SILS in non-healthcare women vs. non-healthcare workers/students.

Health Literacy Tool	All Women	Non-Healthcare Women	Healthcare Workers	Healthcare Students	*p* Value *
	*N* = 7027	*N* = 4036	*N* = 1784	*N* = 1207	
IT-eHEALS (mean ± SD)	26.7 ± 6.8	24.7 ± 6.4	30.2 ± 6.4	28.1 ± 6.2	<0.0001
I-METER n (%)					
Functional HL	2384 (33.9)	714 (17.7)	1146 (64.3)	524 (43.4)	<0.0001
Marginal HL	3925 (55.9)	2751 (68.2)	566 (31.7)	608 (50.4)	<0.0001
Low HL	718 (10.2)	571 (14.1)	72 (4.0)	75 (6.2)	<0.0001
SILS-IT *n* (%)					
High HL	5082 (73.3)	2672 (66.2)	1497 (83.9)	913 (75.6)	
Low HL	1945 (27.7)	1364 (33.8)	287 (16.1)	294 (24.4)	<0.0001

* *p* value (χ^2^ test) refers to comparison between the three groups (non-Wo, HS, and HW).

**Table 4:** Univariate and multivariate analysis on the association between predictors and online health information seeking after medical examination.

Variables	Search Online after Medical Examination N (%)	*p* Value *	Odds Ratio (95%CI)	*p* Value *
Age (Years)		<0.0001		
18–25	1279 (50.5)		Reference	
26–40	1040 (57.8)		1.28 (1.13–1.46)	<0.0001
>40	991 (48.1)		1.00 (0.88–1.14)	ns
Education		0.02		
High school or below	1787 (50.9)		Reference	
Degree	1887 (53.7)		1.07(0.96–1.20)	ns
Presence of chronic illness		<0.0001		
No	3105 (51.0)		Reference	
Yes	569 (60.7)		1.48 (1.27–1.72)	<0.0001
General satisfaction with the healthcare professional		<0.0001		
Very/extremely	2742 (50.0)		Reference	
Moderately	693 (59.3)		1.58 (1.38–1.81)	<0.0001
Not at all/slightly	239 (64.1)		2.04 (1.62–2.57)	<0.0001
Social network health group followers		<0.0001		
No	2237 (47.6)		Reference	
Yes	1437 (61.7)		1.54 (1.38–1.72)	<0.0001
Belief that online information improve own health knowledge		<0.0001		
Not at all/slightly	1001 (39.0)		Reference	
Moderately	1092 (54.0)		1.59 (1.40–1.80)	<0.0001
Very/extremely	1581 (64.9)		2.19 (1.92–2.50)	<0.0001
Belief that online information influence own health habits/decisions		<0.0001		
Not at all/slightly	1173 (40.9)		Reference	
Moderately	989 (56.0)		1.52 (1.34–1.73)	<0.0001
Very/extremely	1512 (63.2)		1.73 (1.52–1.96)	<0.0001
I-METER		ns	Not included	
Functional	1246 (52.3)		-	
Marginal/Low	2057 (52.4)		-	
SILS-IT		<0.0001		
High	2568 (50.5)		Reference	
Low	1106 (56.9)		1.38 (1.22–1.55)	<0.0001
eHEALS score (mean ± SD)				
NOT searching online after medical examination	26.1 ± 7.2		Ref	
Searching online after medical examination	27.3 ± 6.5	<0.0001 **	1.02 (1.0–1.02)	<0.0001

* χ^2^ test was used to calculate *p* value in univariate analysis and logistic regression model in multivariate analysis. ** Mann–Whitney test; ns = not significant.

## Data Availability

The data are stored in a password-protected electronic archive held by the person in charge of the study. Only the person in charge of the study and the researchers of the present paper can access the file archive.

## Supplementary Materials

The following supporting information can be downloaded at: https://www.mdpi.com/article/10.3390/ijerph19084745/s1, Supplementary File S1: Checklist for Reporting Results of Internet E-Surveys (CHERRIES); Supplementary File S2: Online questionnaire.
