# Supplementary material for: Exploring the Online Health Information-Seeking Behavior in a Sample of Italian Women: The “SEI Donna” Study
Source: Int J Environ Res Public Health. 2022 Apr 14;19(8):4745. doi: 10.3390/ijerph19084745 (PMC9024845; doi:10.3390/ijerph19084745)
Supplement: Supplementary file 1 [file ijerph-19-04745-s001.zip › Supplementary File S1_Cherries Checklist.pdf]

Supplementary File S1: Checklist for Reporting Results of Internet E-Surveys (CHERRIES)

Item Category	*Checklist Item*	*Explanation*
**Design**	Describe survey design	The study had a cross-sectional design based on an online self-administered questionnaire. Target population: women aged over 18 years old using the Internet to seek health information.<br>As "open survey" the sample was a convenience sample.
**IRB (Institutional Review Board) approval and informed consent process**	IRB approval	According to the Italian law, this study did not require approval by the Ethics Committee. The protocol was sent to the Ethics Committee of Brescia, only for acknowledgement. Indeed, the study was anonymous at the source and therefore there was no possibility in any way to trace it back to whom filled in the questionnaire.
	Informed consent	Before starting the online questionnaire, a brief description was displayed to each participant containing the following information: the name of the investigators, aim of the study, target population, duration of the survey, data treatment and indication of an email address dedicated to the study to collect any inquiry. Participants could complete the questionnaire only after agreeing to go on.
	Data protection	The study was anonymous at the source. Each participant was identified with a number code. The data have been stored in a password-protected database.
**Development and pre-testing**	Development and testing	The questionnaire was developed based on the scientific literature regarding women's habits and perceptions on online health information. As regards to health literacy we used three validated instruments.<br>The electronic questionnaire was created using LimeSurvey, an advanced online survey software to create quality online surveys.<br>To test the readability, comprehension, and completeness of the questions, the questionnaire was distributed to a small sample of women (n=25) not working in the healthcare field and with different ages.
**Recruitment process and description of the sample having access to the questionnaire**	Open survey versus closed survey	Open survey
	Contact mode	The initial contact with potential participants was made on social media including messaging platforms such as WhatsApp and institutional mailing lists. Other participants could see the link on the website www.ondaosservatorio.it, a website focused on women's health, www.syrio.org the website of Italian Society of Obstetrical-Gynecological-Neonatal Sciences (SYRIO) and the website of an online public health newspaper (www.quotidianosanità.it).
	Advertising the survey	The link to the questionnaire was posted on social media, including public social media pages of the authors of the manuscript, Facebook groups dedicated to women's health, and personal pages of influencers (Instagram, LinkedIn).<br>The survey was also advertised on the website www.ondaosservatorio.it, a website focused on women's health, on official sites of local health units and universities involved in the study, on www.syrio.org the website of Italian Society of Obstetrical-Gynecological-

		Neonatal Sciences (SYRIO) and an online public health newspaper (www.quotidianosanità.it).<br>Mailing lists included mailing lists of all Universities involved in the study, Italian Hygiene Society Regional Sections members, SYRIO members, Regional Health Agency (Tuscany), and patients'/citizens' communities of the Friuli Venezia Giulia Region.<br>In all cases the link was preceded by a brief description of the study and the request for resharing the survey link (snowball sampling)
**Survey administration**	Web/E-mail	The survey link (https://igiene.unibs.it/indagini/index.php/514852?lang=it) was posted on social media pages and websites as previously reported. The same link was sent out via e-mail.
	Context	The website www.osservatorionda.it is the popular website of ONDA, the national observatory for women and gender's health aiming to promote a gender-oriented approach to health, with a special focus on women. The website www.syrio.org is the website of Italian Society of Obstetrical-Gynecological-Neonatal Sciences aiming to disseminate scientific knowledge particularly among health professionals. "Quotidiano sanità" is an online health information newspaper. We referred also to Facebook groups dedicated to different women health topics. We chose different channels (popular and institutional) to avoid recruiting only women studying/working in the health context. In fact, slightly more than half (57%) of the sample was composed by non- healthcare women.
	Mandatory/voluntary	The survey was voluntary.
	Incentives	At the end of the questionnaire, we included a link redirecting to online courses developed by IC-Health (Improving digital health literacy in Europe), a Horizon 2020 project aiming to increase awareness among EU citizens of the opportunities of eHealth tools. These courses designed for general population were available in different languages, including Italian. The access was free. Before starting the survey, potential participants were informed about this possibility.
	Time/Date	The survey link was active from February to July 2019
	Randomization of items or questionnaires	Randomization of items was applied to questions on level of trust in different sources of information, in order to ensure greater attention by the compiler and, thus, a more truthful response.
	Adaptive questioning	We used different adaptive questions or filter questions to reduce complexity of the survey. Some items appeared only in case of responses "yes", "no" or in case of low scores when it was asked to give a response according to a Likert scale.
	Number of Items	There was only a question per page.
	Number of screens (pages)	59
	Completeness check	Limesurvey, the software we used to develop the survey is based on JAVAScript. Specifically, all items were mandatory and appeared red if not completed. JAVAScript was also used to facilitate the response in case of more options. For all items with more options and

		only one response requested, a non-response option was provided.
	Review step	A back button was present.
**Response rates**	Unique site visitor	Not applicable
	View rate (Ratio of unique survey visitors/unique site visitors)	It was not possible to know how many people saw the link and it was not possible to extrapolate the number of unique site visitors also because we used different channels to disseminate the survey link.<br>Anyone who received the questionnaire on the Internet, via email or mobile phone and met the inclusion criteria could fill in it.
	Participation rate (Ratio of unique visitors who agreed to participate/unique first survey page visitors)	92%
	Completion rate (Ratio of users who finished the survey/users who agreed to participate)	70%
Preventing multiple entries from the same individual	Cookies used	Cookies were not used.
	IP check	IP address was not used to guarantee the completely anonymity of the participant.
	Log file analysis	We did not use techniques to exclude multiple entries. Multiple entries may be possible in case of incomplete questionnaires. In fact, these were excluded because of missing data and because they could be due to multiple log in attempts by the same user.<br>In case of complete questionnaires, we excluded the probability to have users who filled out the questionnaire more times. In any case, we thought that this probability was very low due to the different source of dissemination and the length of the questionnaire.
	Registration	Not applicable
Analysis	Handling of incomplete questionnaires	We analyzed only completed questionnaires.
	Questionnaires submitted with an atypical timestamp	The time needed to fill the questionnaire recorded by Limesurvey was mean 16 min. 10 sec. and median, 14 min. 24 sec. We did not exclude questionnaires based on compilation time.
	Statistical correction	We used the techniques for adjustment in observational studies, namely, matching subgroups of sample according to possible confounders. E.g., online Seekers vs non-online seekers; healthcare women vs non-healthcare. Moreover, we carried out a multivariate analysis in order to evaluate all possible confounders.
