# Supplementary material for: Exploring the Online Health Information-Seeking Behavior in a Sample of Italian Women: The “SEI Donna” Study
Source: Int J Environ Res Public Health. 2022 Apr 14;19(8):4745. doi: 10.3390/ijerph19084745 (PMC9024845; doi:10.3390/ijerph19084745)
Supplement: Supplementary file 1 [file ijerph-19-04745-s001.zip › Supplementary File S2_Questionnaire.pdf]

Supplementary File S2: Online questionnaire

In this supplementary materials it is shown the whole questionnaire disseminated via web and social media. Data collected from some questions were not used in this manuscript because we considered that they did not provide information useful for the purposes of the study submitted to the journal. Specifically, questions C2, C3, C8; C13-C23; C28,C30, C32, C43-C49.

Introduction before starting the survey

Dearest, "SEI Donna" is a study carried out by the University of Brescia's Obstetric-Gynecologic-Neonatal sciences departments along with the Public Health and Human Sciences section. Its aim is to study the knowledge, perceptions, and ways of using the Internet, in the context of different issues relating to women's health. Data analysis will allow us to better understand and improve the ways in which information is used with a perspective of health care. The data you provide will be fully confidential and will be used in the statistical analysis for research purposes ONLY. If you are willing to participate in this survey, and allow the use of data you will provide, please click on the "NEXT" button and start filling out the questionnaire. This survey is for female participants over 18 years of age. Completing the questionnaire will take approximately 15 minutes. At the end of the questionnaire, you will find a link redirecting to tools to understand online information search modalities. We thank you in advance for your availability. With thanks for the help in disseminating the questionnaire: AIDM - Associazione Italiana Donne Medico AOUC - Azienda Ospedaliero Universitaria Careggi A.P.E. Onlus - Associazione Progetto Endometriosi Onlus ASL Toscana Centro ATS Val Padana Confartigianato FVG FattoreMamma InfoHandicap di Udine Ordine Medici Provincia FI SISMI (Segretariato Italiano Studenti in Medicina) Società Italiana Igiene - Sezione Toscana

A1. We ask you to give your consent to participate in the study, fully aware that this consent is freely expressed and is revocable anytime during the fill-in process. Keep in mind that, once the questionnaire is sent, it is in no way attributable to the person who filled it in and, therefore, we will not be able to delete the given data, if requested. If you have any doubts o need further information you can reach us at the following email address: seidonna@unibs.it

Do you consent to participate in the "SEI Donna" study? (YesNo)

Section	Questions
**Inclusion criteria***	**A2. Gender: (**F/M)<br>**A3. Age:**<br>**A4. Have you ever used/Do you use the internet to search for health-related information?** (Yes/No)
**Sociodemographic information**	**B1. Education level:**<br>Elementary education<br>Secondary school education<br>High school education<br>University degree<br>**B2. Nationality:**<br>Italian<br>European<br>Extra-European<br>**B3. Residence (region):**<br>**B4. Do you study or work in the healthcare sector?** (Yes/No)<br>**B5. Occupation:**<br>Student<br>Housewife<br>Employee

	Freelancer<br>Unemployed<br>Retired<br>**B6. Marital status:**<br>Single<br>Cohabitant/Married<br>Separated/Divorced<br>Widow
**Web and social media use and perception**	**C1. Among the following websites, which one do you consider most trustworthy:**<br>www.dolceattesa.it<br>www.uppa.it<br>www.salute.gov.it<br>www.centropsichedonna.it<br>www.donnamoderna.com/salute<br>www.donnainsalute.it<br>None of the above<br>I am not able to assess<br>**C2. When you wish to search for health-related information on the Internet, how do you normally carry out the task? (**Select up to 3 answers IN ORDER OF FREQUENCY, dragging them in the box to the right)[a]<br>I directly look up Wikipedia<br>I directly look up non-institutional thematic websites that I know<br>I directly look up social media (e.g. Facebook, Twitter, Instagram, etc.)<br>I type in a keyword (e.g. menopause) on a search engine (e.g. Google, Yahoo, etc.) and I choose a website among the results<br>I directly look up institutional websites (e.g. Ministry of Health, Italian National Institute of Health, etc.)<br>**C3. In your opinion, why do certain websites appear in the first position in a search engine? NB: Do not take into account websites that appear in the advertisement section, i.e. those preceded by "Adv.", as per the example:**<br>Because they include the most trustworthy information<br>They are there by chance<br>They are the most clicked on<br>They represent the result of the PageRank algorithm for the specific search engine<br>They are those that payed the most to be in the first positions<br>I do not know<br>**C4. On social media, do you follow any group dealing with health-related information?** (Yes/no)<br>**C5. Which group dealing with health information on social media do you follow?** If you selected at least one social medium, please write in the space below the name of the group<br>Facebook ______<br>Twitter _______<br>Instagram _____<br>Linkedln ______<br>WhatsApp _____<br>Other ______<br>**C6. Do you follow blogs and/or communities that deal with health-related information?** (Yes/No)<br>**C7. Which blog and/or communities related to health information?**<br>**C8. Do you follow websites for associations of patients affected by specific health problems?** (Yes/No)

	**C9. As regard to health, what kind of information do you look for the most on the Internet? (**Select up to 3 answers IN ORDER OF FREQUENCY, dragging them in the box to the right.)[a]<br>Personal care/beauty<br>Purchase of health related products<br>Alternative medicine (e.g. homeopathy, acupuncture, phytotherapy)<br>Cosmetic surgery or medicine<br>Lifestyle (e.g. nutrition, physical activity, alcohol, smoking)<br>Specific illnesses or conditions (e.g. stomachache)<br>Therapies or procedures (how to take medications, what does a specific surgery consists of)<br>Specialists, hospitals, or clinics
**Sociodemographic information**	**C10. Are you pregnant at the moment?** (Yes/No)<br>**C11. Do you have children?** (Yes/No)<br>**C12. Do you have pediatric aged children (0-14)?** (Yes/No)<br>**C13. Are you in menopause?** (Yes/No)
**Web and social media use based on phases of a woman's life**[b]	**C14. Have you ever looked for information on fertility/contraception online?** (Yes/No)<br>**C15. As for fertility/contraception, what kind of information have you mostly looked for?** (Select up to 3 answers IN ORDER OF FREQUENCY, dragging them in the box to the right.)[a]<br>Contraceptive methods<br>Lifestyle (nutrition, alcohol, smoking, physical activity)<br>Voluntary termination of pregnancy<br>In fertility/sterility<br>Menstrual cycle and ovulation<br>Assisted reproduction<br>None of the above<br>**C16. Have you ever looked for information on pregnancy online?** (Yes/No)<br>Note: This question appeared only for pregnant women<br>**C17. As for pregnancy, what kind of information have you mostly looked for?** (Select up to 3 answers IN ORDER OF FREQUENCY, dragging them in the box to the right.)[a]<br>Specialists, hospitals, or clinics<br>Infectious disease prevention (rubella, toxoplasmosis, etc.)<br>Supplements<br>Pregnancy and work<br>Car safety<br>Oral health<br>Drug safety<br>Periodic monitoring (blood tests, ultrasounds, gynecological examinations)<br>Symptoms (e.g. nausea, vomit)<br>Nutrition<br>Physical activity<br>Sexuality<br>Pregnancy-related conditions (Gestational Diabetes, preeclampsia)<br>Pre-birth courses<br>Vaccinations during pregnancy<br>None of the above<br>**C18. Have you ever looked for information on childbirth on the internet?** (Yes/No)<br>**C19. As for childbirth, what kind of information have you mostly looked for?** (Select up to 3 answers IN ORDER OF FREQUENCY, dragging them in the box to the right.)[a]

	Storage and donation of neonatal cord blood<br>Lotus birth<br>Spontaneous delivery<br>Cesarean birth<br>Painless childbirth (natural of pharmacologically induced)<br>Other childbirth modalities (e.g. water birth)<br>Induction of labor<br>Placenta/Afterbirth<br>None of the above<br>**C20. Have you ever looked for information on infants on the internet?** (Yes/No)<br>**C21. As for the infant, what kind of information have you mostly looked for?** (Select up to 3 answers IN ORDER OF FREQUENCY, dragging them in the box to the right.)[a]<br>Skin-to-skin contact with the newborn<br>Hygienic care (e.g. baths, care and loss of the umbilical cord)<br>Brestfeeding<br>Newborn massage<br>Complementary feeding (e.g. formula)<br>Neonatal care products<br>Car safety<br>Weaning<br>Symptoms and/or neonatal illnesses<br>Protected discharge (continuity of care in the territory)<br>Healthcare professionals (e.g. Pediatrician, obstetric)<br>Other non-healthcare professionals<br>Recognition and registration of the newborn at the Registry Office<br>None of the above<br>**C22. Have you ever looked for information on menopause on the internet?** (Yes/No)<br>**C23. As for menopause, what kind of information have you mostly looked for?** (Select up to 3 answers IN ORDER OF FREQUENCY, dragging them in the box to the right.)[a]<br>Alteration of menopause (e.g. late or early menopause)<br>Symptoms (e.g. hot flushes, night sweats)<br>Oral health<br>Mood<br>Sleep<br>Physical activity<br>Sexuality<br>Osteoporosis prevention<br>Hormonal therapies<br>Breast cancer screening<br>Beauty products<br>None of the above
**Level of trust in source of information**	**C24. How much do you trust heath related information given to you by specialist doctors (e.g. gynecologist, cardiologist, etc.)?**<br>On a scale of 1 (not at all) to 7 (a lot) or I have never been seen by a specialist doctor
**Web and social media use**	**C25. Have you ever bought products for healthcare or well-being online?** (Yes/No)<br>**C26. What kind of healthcare/well-being products have you purchased online?** Write down below, if any, which products are most frequently purchased.<br>Cosmetics and body care products<br>Medications<br>Herbal products

	Homeopathic products<br>Sex-related products<br>Products for infant care and well-being<br>Contraceptives<br>Pregnancy tests<br>Ovulation tests<br>Supplements<br>Other
**Level of trust in source of information**	**C27. How much do you trust heath related information shared by friends through social media (e.g. Facebook, Twitter, WhatsApp, etc.)?**<br>On a scale of 1 (not at all) to 7 (a lot)<br>**C28. As for social media, in the event you were not completely trusting in the received health information, could you address the reasons why?**[c]<br>**Information was:**<br>(Select up to 3 answers IN ORDER OF IMPORTANCE, dragging them in the box to the right.)[a]<br>Incomplete, not detailed enough<br>Useless, inadequate to my need<br>Difficult to understand<br>Aimed at the purchase of products<br>Not transparent enough, with possible conflict of interest<br>None of the above<br>**C29. How much do you trust heath related information shared by NON-INSTITUTIONAL WEBSITES?**[c]<br>On a scale of 1 (not at all) to 7 (a lot)<br>**C30. As for NON-institutional websites, in the event you were not completely trusting in the received health information, could you address the reasons why?**<br>(Select up to 3 answers IN ORDER OF IMPORTANCE, dragging them in the box to the right.)[a]<br>**Information was:**<br>Incomplete, not detailed enough<br>Useless, inadequate to my need<br>Difficult to understand<br>Aimed at the purchase of products<br>Not transparent enough, with possible conflict of interest<br>None of the above
**Web perception**	**C31. Do you think that information obtained through the Internet improve your knowledge on health-related matters?**<br>On a scale of 1 (not at all) to 7 (a lot)
**Level of trust in source of information**	**C32. How much do you trust heath related information shared by INSTITUTIONAL WEBSITES?**[c]<br>On a scale of 1 (not at all) to 7 (a lot)<br>**C33. As for institutional websites, in the event you were not completely trusting in the received health information, could you address the reasons why?**[b]<br>**Information was:**<br>(Select up to 3 answers IN ORDER OF IMPORTANCE, dragging them in the box to the right.)[a]<br>Incomplete, not detailed enough<br>Useless, inadequate to my need<br>Difficult to understand<br>Aimed at the purchase of products<br>Not transparent enough, with possible conflict of interest

	None of the above
**Web perception**	**C34. How much do you think information acquired online have influenced your health-related decisions/habits?** E.g., Choosing the GP, of a product, joining of a certain screening program, lifestyle change<br>On a scale of 1 (not at all) to 7 (a lot)
**Level of trust in source of information/ satisfaction with healthcare professional**	**C35. How much do you trust health related information provided by your GP?**<br>On a scale of 1 (not at all) to 7 (a lot)<br>**C36. How much do you trust health related information provided by your obstetrician?**<br>On a scale of 1 (not at all) to 7 (a lot) or I have never spoken to an obstetrician<br>**C37. On a general level, considering every time you have made contact with a healthcare professional, how satisfied would you consider yourself?**<br>On a scale of 1 (not at all) to 7 (a lot)<br>**C38. As for healthcare professional, thinking of all times you were not satisfied with the interaction, what were the most prominent problems?**[c]<br>(Select up to 3 answers IN ORDER OF IMPORTANCE, dragging them in the box to the right.)[a]<br>Time for the consultation<br>Competence of the healthcare worker<br>Clarity of language<br>Availability to clarify/elaborate on the topic<br>Ability to listen to my needs<br>Lack of empathy with regards to the discussed topic<br>None of the above
**Web and social media use**	**C39. Have you ever used the Internet/social media to gather further information after a doctor's/obstetrician's appointment?** (Yes/No)<br>**C40. If yes, why?**<br>(Select up to 3 answers IN ORDER OF IMPORTANCE, dragging them in the box to the right.)[a]<br>Information provided were not detailed enough<br>I wanted to learn more on the topic<br>The healthcare worker suggested me so<br>I did not trust what had been told me<br>I wanted moral support to face my health problem<br>It did not seem like the healthcare worker cared about my health problem<br>None of the above<br>**C41. In the event you have used the internet/social media to gather further information following a doctor's/obstetrician's appointment, what did you use it or for what information?**<br>(Select up to 3 answers IN ORDER OF IMPORTANCE, dragging them in the box to the right.)[a]<br>To understand test results<br>For the prescribed therapy (e.g. medicines, possible side effects)<br>For possible alternative cures<br>To gather accounts from other people's experiences<br>Possibility to connect with other specialists and/or hospitals<br>None of the above
**Health profile**	**C42. In general, your health is:**<br>On a scale of 1 (poor) to 7 (excellent)<br>**C43. Do you regularly undergo oncologic screenings? E.g. pap test, breast examination** (Yes/No)

	**C44. On average, how many servings of fruit and vegetables do you consume on a daily basis?**<br>4-5 servings<br>3 servings<br>Less than 3 servings<br>I do not eat fruit/vegetables on a daily basis<br>**C45. Do you regularly exercise?** (Yes/No)<br>**C46. How many days a week do you exercise?** (From 1 to 7)<br>**C47. Are you a smoker?** (Yes/No)<br>**C48. Do you consume alcoholic beverages?**<br>Yes, everyday<br>Yes, occasionally<br>No<br>**C49. How much do you think each of the following behaviors influence your lifestyle?**<br>On a scale from 1 (not at all) to 7 (very much)<br>Following a healthy diet _____<br>Schedule screening tests _____<br>Avoid smoking ____<br>Exercise regularly ____<br>Avoid excessive consumption of alcoholic beverages ____<br>**C50. Do you suffer from chronic diseases?** E.g., Diabetes mellitus, rheumatoid arthritis, cancer (Yes/No)
**Health literacy**	**C51. I-METER test (see reference [22])**<br>**C52. How many times do you require help reding instructions, leaflets, or other materials that was given to you by your doctor, pharmacist, or other healthcare worker? (SILS-IT)**<br>Never<br>Rarely<br>Sometimes<br>Often<br>Always
**e-Health Literacy**	**C53. How much do you agree with the following statements, on a scale from 1 (strongly disagree) to 5 (strongly agree)?**<br>I know how to find helpful health resources on the Internet<br>I know how to use the Internet to answer my health questions<br>I know what health resources are available on the Internet<br>I know where to find helpful health resources on the Internet<br>I know how to use the health information I find on the Internet to help me<br>I have the skills I need to evaluate the health resources I find on the Internet<br>I can tell high quality from low quality health resources on the Internet<br>I feel confident in using information from the Internet to make health decisions

Note:

*In case of response "M" or age "<18 years" the survey stopped. In case of response "No" to question A4 survey stopped after the compilation of sociodemographic information.

[a]Respondents had the possibility to drag the option selected in a box to the right of the screen

[b]Questions C16,18, 20 and 22 were displayed in case of "Yes" response to C10, C11, C12, C13 respectively. Questions C15,17,19,21 and 23 were displayed only in case of "Yes" response at the previous questions.

[c]Question was displayed for respondents who gave a score less than 6
